# Immunomodulatory cytokines: directing and controlling immune activation

**DOI:** 10.1186/ar3418

**Published:** 2011-09-16

**Authors:** Fillatreau Simon

**Affiliations:** 1 Deutsche Rheuma-Forschungszentrum, a Leibniz institute. Chariteplatz 1, 10117 Berlin, Germany

## 

A hallmark of immunity is the production of a multi-faceted array of inflammatory cytokines that exerts decisive influence on innate and adaptive immune responses. The importance of these mediators of intercellular communication in autoimmunity is illustrated by the beneficial effects resulting from blockade of single cytokines such as TNF or IL-6 in these diseases. B lymphocytes can also play pathogenic roles during autoimmune disease because B cell depletion often led to amelioration of disease in patients treated with rituximab [1]. The pathogenic functions of B cells during autoimmune diseases are poorly understood. They might involve autoantibody production, yet the beneficial effects resulting from the depletion of B cells usually preceded reduction in autoantibody titers [2]. We recently demonstrated that the pathogenic activities of B cells during experimental autoimmune encephalomyelitis (EAE), the primary animal model for multiple sclerosis (MS), were largely mediated through provision of inflammatory cytokines. B cells from MS patients also produced enhanced amounts of inflammatory cytokines, compared to B cells from healthy individuals, and this abnormality was corrected through B cell depletion i.e. B cells returning at 1 year after rituximab treatment showed a normalized cytokine response. Inflammatory processes are usually balanced by counter-regulatory circuits involving anti-inflammatory cytokines such as interleukin (IL)-10 [3,4]. We previously demonstrated that IL-10 production by B lymphocytes played a determinant role for the resolution of EAE [5]. Accordingly, IL-10 might provide a powerful means for controlling pathogenic immune reactions. However, administration of IL-10 into patients achieved little beneficial effects in the clinic, asking for a better understanding of the immunosuppressive biology of this molecule. To this end, we pursued the characterization of IL-10-producing B cells in a model of infection by the intracellular bacterial pathogen *Salmonella typhimurium*. Using a novel strain of IL-10.eGFP knock-in mice to facilitate the tracking of these cells, we could identify IL-10 producing B cells already within 24 hours after infection in spleen, and demonstrated that all IL-10^+ ^B cells expressed the cell surface receptor CD138, which is a distinctive marker of antibody secreting cells [6,7]. IL-10 expression was undetectable in other cell types such as dendritic cells, macrophages, or T cells at this stage, implying that plasmablasts were the first producers of IL-10 during immune reactions. These data suggest that IL-10 might be needed at a very early stage of immune reactions to be suppressive. Collectively, our data showed that B cells have a dual role during autoimmune diseases, acting both as drivers and regulators of pathogenesis, and identified cytokine production as core mechanisms in these complex functions.
